# Dimensions of Mindfulness and Their Relations with Psychological Well-Being and Neuroticism

**DOI:** 10.1007/s12671-016-0645-2

**Published:** 2016-11-29

**Authors:** Luca Iani, Marco Lauriola, Valentina Cafaro, Fabrizio Didonna

**Affiliations:** 1 Department of Human Sciences, European University of Rome, Via degli Aldobrandeschi 190, 00163 Rome, Italy; 2 Department of Social and Developmental Psychology, University of Rome ‘Sapienza’, Rome, Italy; 3 Unit for Mood & Anxiety Disorders, Department of Psychiatry, Casa di Cura Villa Margherita, Arcugnano, Vicenza, Italy

**Keywords:** Mindfulness, Psychological well-being, Subjective well-being, Neuroticism, Mediation, Five-facet mindfulness questionnaire

## Abstract

In this study we examined whether differences in the habitual use of mindfulness skills were associated with specific well-being and neuroticism aspects. Two hundred eleven volunteers aged 21–84 years completed measures of mindfulness, neuroticism, psychological well-being (PWB), and subjective well-being (SWB). Describing, observing, and acting with awareness (i.e., the mindfulness “what” skills) were positively correlated with personal growth, purpose in life, and autonomy (i.e., the “core” eudaimonic components of PWB). Nonreactivity and nonjudging (i.e., the mindfulness “how” skills) were negatively associated with neuroticism aspects, such as withdrawal (e.g., depression) and volatility (e.g., anger). Describing and nonreactivity were the only mindfulness skills significantly correlated with the SWB measures. Acting with awareness mediated the effect of both withdrawal and volatility on eudaimonic well-being outcomes. Describing had consistent mediation effects across all well-being measures, but only for the withdrawal aspect. Nonreactivity and nonjudging did not mediated withdrawal when considering eudaimonic well-being as outcomes. Mediation effects for nonjudging and nonreactivity were found between volatility and SWB markers as well as between volatility and self-acceptance, environmental mastery, and positive relations with others (i.e., the “other” eudaimonic PWB components). In sum, the mindfulness “what” skills were important for eudaimonic well-being, especially for internalizing individuals. Authors discuss the usefulness of a facet-level analysis of mindfulness for examining incremental validity of some facets over others in accounting for different well-being outcomes measures. Clinical implications are also discussed.

## Introduction

Is mindfulness related to well-being and neuroticism? A growing body of studies indicates that the answer is overall positive. But what kind of relation is more theoretically meaningful and eventually useful for its clinical implications? Research has considered the relationships of mindfulness with neuroticism and well-being based on total scores or broad trait measures. However, a recent trend has started looking at these relationships using either mindfulness facets with broad neuroticism, or mindfulness total score with neuroticism facets, or mindfulness facets with psychological well-being (PWB) total score. A facet-level analysis for all three constructs might shed light on unique relationships that tend to get overlooked otherwise. Moreover, the mindfulness facets might also be considered as intervening variables in the robust empirical association between neuroticism and well-being.

The link between neuroticism and well-being has been clearly established in recent years (Steel et al. 2008). Less is known about *how* neuroticism might influence one’s well-being. Neuroticism is not easily changeable and its mean level remains stable over 40 years of age (Roberts et al. 2006). However, mindfulness might represent a process through which neuroticism lead to well-being. Therefore, considering mindfulness as potential mediator in the relationship between neuroticism and well-being would be fruitful for theoretical and clinical purposes (Hampson 2012).

In the past decades, advances in clinical psychology interventions have borrowed and adapted to secular context the old concept of mindfulness meditation. Mindfulness is defined as “paying attention in a particular way: on purpose, in the present moment, and nonjudgmentally. This kind of attention nurtures greater awareness, clarity and acceptance of present-moment reality” (Kabat-Zinn 1994, p. 4). It is a human natural capacity and also a set of skills that can be cultivated and developed through a regular meditation practice or specifically tailored interventions (Baer et al. 2006; Linehan 2014).

After an initial interest in clinical change, the literature has been focused on the assessment of mindfulness skills. There is an ongoing debate on the measurement of mindfulness and its specific qualities. In particular, phenomenological understanding of mindfulness and meditation cannot exclusively depend on intellectual knowledge or on scientific methods of assessment (Grossman and Van Dam 2011). An exploration of one’s own subjective experience could integrate the intellectual and conceptual categories used by scientists to study the characteristics and qualities of mindfulness. Scientific literature, however, has proposed a number of psychometric scales relying on similar operational definitions of the construct. These measures popularized the concept that mindfulness is a multifaceted construct. A facet-level analysis of mindfulness skills is deemed important for examining incremental validity of some facets over others in predicting different outcomes (Baer et al. 2006). The use of multifaceted mindfulness measures has also contributed to strengthen the evidence that support the effectiveness of mindfulness-based interventions (e.g., Haenen et al. 2016).

In order to develop a comprehensive assessment instrument, the Five Facets Mindfulness Questionnaire (FFMQ) was created (Baer et al. 2006). The 39-item FFMQ was derived from an exploratory factor analysis of a comprehensive set of mindfulness questionnaires. The analysis yielded the following five factors: (1) nonreactivity to inner experience, (2) observing, (3) acting with awareness, (4) describing, (5) nonjudging of experience. Subsequent studies have shown that the proposed five-factor structure of mindfulness was empirically robust, being cross-validated in clinical and nonclinical samples, as well as in different countries and languages (Lilja et al. 2011; Veehof et al. 2011). In order to provide a more manageable instrument to be easily used for clinical research purposes, a 24-item short form (FFMQ-SF) was recently developed (Bohlmeijer et al. 2011). The analysis yielded a factor structure consistent with the longer version: observing or “noticing or attending to internal and external experiences”; describing or “labeling internal experiences with words”; acting with awareness or “attending to one’s activities of the moment”; nonjudging of inner experience or “taking a nonevaluative stance toward thoughts and feelings”; nonreactivity to inner experience or “allowing thoughts and feelings to come and go, without getting caught up in or carried away by them” (Bohlmeijer et al. 2011, p. 309).

Many psychological interventions have been developed to enhance mindfulness skills (Didonna 2009). For instance, Dialectical Behavior Therapy (DBT) targets both the “what” skills and the “how” skills of mindfulness (Linehan 2014). The mindfulness “what” skills encompass observing, describing, and participating with awareness. These skills are typically used when “one is learning new behaviors, when there is some sort of problem, or when a change is necessary or desirable” (Linehan 2014, 154). The mindfulness “how” skills “are about *how* one observes, describes, and participates; they include taking a nonjudgmental stance (‘nonjudgmentally’), focusing on one thing in the moment (‘one-mindfully’), and doing what works (‘effectively’)” (Linehan 2014, 154).

Although breaking down mindfulness in facets could lead to an oversimplified view (Grossman and Van Dam 2011), mindfulness-based therapies, such as DBT and others, need and use skills-based protocols to implement the treatments and use multi-faceted assessment scales to measure their effectiveness (Linehan 2014). Recent studies have shown that intervention protocols based on the “what” and the “how” skills reduced mental health problems (e.g., anxiety and depression) and improved one’s psychological resources (e.g., self-esteem) (e.g., Erb et al. 2013; Paulik et al. 2010). These results suggest that different facets of mindfulness may act differently in clinical settings and that using a total score of mindfulness (i.e., a broad level of analysis) might be misleading. For instance, only some facets might be involved as predictors of health outcomes in clinical trials (e.g., Forman et al. 2007; Haenen et al. 2016).

Neuroticism is the “tendency to experience negative, distressing emotions and to possess associated behavioral and cognitive traits” (Costa and McCrae 1987, p. 301). As such, neuroticism is hierarchically organized with a general factor at the top, narrow facets at the bottom (i.e., anxiety, anger, depression, self-consciousness, immoderation, and vulnerability; Johnson 2014), and intermediate aspects between the two levels (i.e., “withdrawal” and “volatility”; DeYoung et al. 2007).

Anxious individuals tend to be tense, fearful, worried, apprehensive, nervous, and jittery. Angry individuals typically experience anger, frustration, and bitterness. Depressive individuals tend to be hopeless, guilty, sad, lonely, downhearted, and blue. Self-conscious individuals typically experience shame and embarrassment and tend to feel inferior and uncomfortable when they are the center of attention. Immoderate (i.e., impulsive) individuals are unable to control and resist cravings and urges (e.g., for cigarettes, food, possessions), hasty, and sarcastic. Vulnerable individuals tend to be unable to deal with stress, easily rattled, hopeless, dependent, and panicked (Johnson 2014). Depression, anxiety, and vulnerability are markers of “withdrawal,” which accounts for augmented stress reactivity as well as for negative affect directed inward. Instead, “volatility” encompasses anger and immoderation, namely irritability, emotional lability, and failure to inhibit emotional impulses. The literature has linked withdrawal and volatility with internalized and externalized psychopathology, respectively (e.g., Ormel et al. 2005).

As it regards well-being, different concepts have gained an important role in scientific literature (e.g., psychological or eudaimonic well-being, and subjective or hedonic well-being; Huta and Waterman 2014). On the one hand, subjective well-being (SWB) “is a broad category of phenomena that include people’s emotional responses, domain satisfactions, and global judgments of life satisfaction” (Diener et al. 1999, p. 277). As such, hedonia includes pleasure, enjoyment, satisfaction, and reduced distress (Huta and Waterman 2014). On the other hand, PWB is defined as the “perception of engagement with existential challenges of life” (Keyes et al. 2002, p. 1007), and it refers to one’s sense of growth and human fulfillment, to how people strive to realize their true potential (Ryff 2014). The trait level correlations between eudaimonic and hedonic measures ranged from 0 to .4 when considering them as orientations, and from .3 to .6 when they were conceptualized as experiences (Huta and Waterman 2014). In sum, eudaimonia and hedonia are distinct concepts, both theoretically and empirically, although they often co-occur (Huta 2015).

Getting back to the PWB measure, we refer to autonomy, personal growth, and purpose in life as the “core” eudaimonic components of PWB (Bauer et al. 2005; Ryff and Keyes 1995); instead, we refer to self-acceptance, environmental mastery, and positive relations with others as the “other” eudaimonic components. In fact, there is some evidence that the former components were weakly correlated to hedonic measures of well-being; instead, the latter were mildly or moderately correlated to SWB measures (Ryff and Keyes 1995).

The correlations between neuroticism and mindfulness have been recently established at a broad trait level (e.g., the effect size *r* was .45; Giluk 2009). At a facet-level analysis, mild negative correlations of neuroticism with describing and nonreactivity, and moderate negative correlations with acting with awareness and nonjudging have been found (Baer et al. 2006). Instead, observing was not at all related to neuroticism. Other studies also showed that specific withdrawal facets, such as depression, self-consciousness, and vulnerability, were more strongly associated with a global mindful attention awareness score than other facets like anger and immoderation (e.g., Brown and Ryan 2003). Likewise, there is evidence of negative relationships between the attention awareness score and impulsiveness as well as angry hostility (Brown and Ryan 2003). To our knowledge, the links of mindfulness skills with neuroticism intermediate aspects have not yet been extensively studied, although this issue might have important implications for targeting clinical interventions. For instance, acting with awareness, nonreactivity, and nonjudging were the most robust predictors of both depression and worry symptoms (Barnes and Lynn 2010; Fisak and Von Lehe 2012). Therefore, research has suggested that targeting these three mindfulness facets could reduce withdrawal tendencies and their relationships with well-being.

The relationship between neuroticism and well-being has been extensively examined (e.g., the effect sizes *r* were −.46, −.37, and −.29 for happiness, life satisfaction, and positive affect, respectively; Steel et al. 2008). Recent studies have investigated the direct effect of neuroticism facets on well-being outcomes (Albuquerque et al. 2012; Anglim and Grant 2016). These studies showed that facets were more useful than broad traits in accounting for SWB, and that depression and vulnerability were unique incremental predictors.

Mindfulness is also linked to well-being (e.g., the effect size *r* was .34 for positive affect; Giluk 2009). All mindfulness facets (but observe) moderately correlated with PWB total score (e.g., Baer et al. 2008). Furthermore, PWB and SWB were significantly correlated with mindfulness at the broad level of analysis (Brown and Ryan 2003). Thus, a comprehensive study of mindfulness and well-being should investigate the relations of specific mindfulness skills with both SWB and PWB measures.

**Fig. 1:**
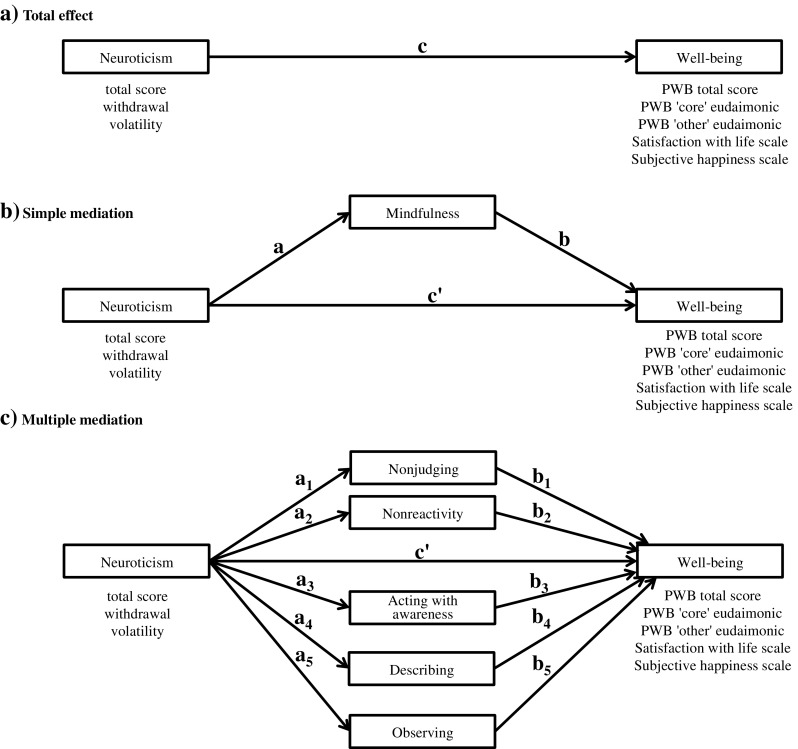
Conceptual models tested in the study. *c* = total effect that links neuroticism to well-being; *a* = effect of neuroticism on mindfulness; *b* = effect of mindfulness on well-being; *a*_*1*_–*a*_*5*_ = effects of neuroticism on mindfulness facets; *b*_*1*_–*b*_*5*_ = effects of mindfulness facets on well-being; *c′* = direct effect of neuroticism on well-being after controlling for mindfulness. Outside the boxes are specific aspects of neuroticism and well-being tested in the analyses

There is a need to examine the relationship between neuroticism, mindfulness, and well-being at the facet-level, and investigate the mediating role of mindfulness in the neuroticism’s link to well-being. In cross-sectional studies with mediational hypothesis, theory is important to justify a particular ordering of variables. Neuroticism is deemed a stable personality trait, especially during adulthood. By contrast, mindfulness, as a set of skills, can be trained through regular meditation or modified through specific mindfulness and acceptance-based interventions. In our conceptual model, mindfulness can be viewed as an intervening variable accounting for the relation between neuroticism and well-being (see Fig. 1).

The aim of the present study was twofold. First, to investigate how different mindfulness facets were differently and uniquely associated with neuroticism and well-being aspects. Second, to assess whether different mindfulness skills might be the mechanisms by which withdrawal and volatility were related to hedonic and eudaimonic well-being. We addressed these research questions through examining the correlations carried out at different levels of analysis and testing mediation models.

## Method

### Participants

As a part of a larger study (Lauriola and Lani 2016), the data were collected from 211 adults (72% females, *M*_age_ = 56.4 years, age range 21–84 years) attending courses at the Popular University of Rome (Upter). As seen in Table 1, the majority of participants was retired (52.3%) and had a Master’s degree (60.5%).

**Table 1:** Sample descriptive statistics

	Males		Females		Total	
	*n*	%	*n*	%	*n*	%
Age category						
21–39	8	15.7	25	21.2	33	19.5
40–59	7	13.7	40	33.9	47	27.8
60–69	18	35.3	34	28.8	52	30.8
70–84	18	35.3	19	16.1	37	21.9
Marital status						
Married	33	58.9	66	46.8	99	50.3
Never married	10	17.9	42	29.8	52	26.4
Separated/divorced/widowed	13	23.2	33	23.4	46	23.4
Education level completed						
Junior high school	4	7.1	2	1.4	6	3.1
Senior high school	20	35.7	51	36.7	71	36.4
College	32	57.1	86	61.9	118	60.5
Employment status						
Employed	17	30.4	40	28.0	57	28.6
In search of employment	0	0.0	5	3.5	5	2.5
Housewife	0	0.0	8	5.6	8	4.0
Student	2	3.6	4	2.8	6	3.0
Retired	33	58.9	71	49.7	104	52.3
Temporary worker	1	1.8	9	6.3	10	5.0
Other/unspecified	3	5.4	6	4.2	9	4.5

### Procedure

Participants individually completed the paper-and-pencil versions of the instruments in a quiet and comfortable room, before or after regular class hours. Forty-two participants refused to participate in the study. The study was approved by the ethical review board for psychological research of the European University of Rome and it was in accordance with the 1964 Helsinki declaration. Verbal consent was obtained from all participants before data collection, following detailed explanations as to the aim of the study, the voluntary nature of participation, and the right to withdraw from the study at any moment. Moreover, participants were informed that all data were anonymized at source. The questionnaires were collected over a 3-week period.

## Measures

### Mindfulness

The Italian short form of the Five Facet Mindfulness Questionnaire (FFMQ-SF, 24 items; Bohlmeijer et al. 2011) was used to assess major aspects of mindfulness skills: observing, describing, acting with awareness, nonjudging, and nonreactivity. Items are measured on a five-point Likert scale, ranging from 1 (*never or very rarely true*) to 5 (*very often or always true*). The FFMQ-SF was translated into Italian by the first two authors and back-translated by a native English speaker. Comparison of the back-translation with the original English version revealed no discrepancies. Cronbach’s *α* were similar to those of the original version for all mindfulness skills except for describing, whose *α* was lower in our sample (*α* = .67, .76, .81, .77, and .77 for describing, observing, acting with awareness, nonreactivity, and nonjudging, respectively). Reliability coefficient for the mindfulness total score was .72.

### Trait Neuroticism

Neuroticism, as defined by six facets, was assessed using 24 items in Italian language from the International Personality Item Pool (IPIP-NEO; Johnson 2014). For each item, participants reported the extent to which each statement describes them, from 1 (*Very inaccurate*) to 5 (*Very accurate*). Translation and back-translation procedures for the neuroticism facets were similar to those adopted for the FFMQ-SF. Though not previously formally validated in Italian language, the scales have been used in large cross-sectional studies (Lauriola and Lani 2016). In the present study, Cronbach’s *α* were .72, .80, .62, .46, .62, and .72 for anxiety, anger, depression, self-consciousness, immoderation, and vulnerability, respectively. Reliability coefficients were slightly lower than those of the original English version for anxiety, anger, and vulnerability, and lower for immoderation, depression, and self-consciousness. Cronbach’s *α* coefficients were .81, .80, and .59 for total score, withdrawal, and volatility, respectively.

### Subjective Well-Being Measures

Life satisfaction was measured using an Italian version of the five-item Satisfaction With Life Scale (SWLS; Diener et al. 1985). Items are rated on a seven-point Likert scale (1 = *strongly disagree*, 7 = *strongly agree*). The Italian version of the SWLS has been used previously in large cross-sectional studies (e.g., Lauriola and Lani 2016), though not formally validated. Cronbach’s *α* was .89 in the present sample, and it was adequately comparable to that one of the original English version.

Subjective happiness was assessed using the Italian validated version of the four-item Subjective Happiness Scale (SHS; Iani et al. 2014; Lyubomirsky and Lepper 1999). Cronbach’s *α* was lower than that one of the original English version (.73 in this sample).

### Psychological Well-Being

The 18-item Italian validated version of the Psychological Well-Being Scales was used to measure six facets of PWB: self-acceptance, positive relations with others, autonomy, environmental mastery, purpose in life, and personal growth (Ruini et al. 2003; Ryff and Keyes 1995; Sirigatti et al. 2013). Items are rated on a six-point Likert scale (1 = *strongly disagree*, 6 = *strongly agree*). In the present study, Cronbach’s *α* were greater than those of the original English version (*α* = .78, .55, .51, .72, .56, and .52 for self-acceptance, autonomy, environmental mastery, personal growth, positive relations with others, and purpose in life, respectively). Reliability coefficient for the PWB total score was .81.

## Data Analysis

The relationships between FFMQ-SF and other psychological variables were assessed by standard Pearson correlation. The interpretation of the magnitude of correlation coefficients was based on Cohen’s guidelines (1988). Separate factorial ANOVAs were conducted on FFMQ-SF facets total scores, considering gender, age (<60 vs ≥60 years), and education level (high school vs college graduate) as between factors.

Mediation analyses were carried out using the SPSS PROCESS macro. A bootstrapping procedure (with 10,000 bootstrap samples) to estimate 95% confidence intervals (95% CI) was used. A 95% CI that does not include zero provides evidence of a significant indirect effect (Hayes 2013). First, simple mediation models were carried out for mindfulness total score as mediator of the relationships between neuroticism (i.e., total score, withdrawal, and volatility) and well-being measures (PWB total score, “core” and “other” eudaimonic PWB components, SWLS, and SHS). Then, multiple mediation models included the mindfulness facets as mediators of the aforementioned relationships. According to our conceptual model, significant indirect effects in multiple mediation analysis provide support for the unique role of each specific facet as mediator.

## Results

Describing was moderately and positively correlated with autonomy, mildly and positively correlated with purpose in life and personal growth. Acting with awareness was mildly and positively correlated with purpose in life. Observing was mildly and positively correlated with personal growth. Observing, describing, and acting with awareness were the only facets associated with the “core” eudaimonic PWB components. Moreover, observing had no significant correlations with self-acceptance, environmental mastery, and positive relations with others (i.e., the “other” eudaimonic PWB facets). Nonreactivity and nonjudging were also less correlated with the PWB facets. In particular, nonjudging was not correlated with the “core” eudaimonic PWB components, as well as with self-acceptance.

Nonreactivity was mildly and positively correlated with both happiness and life satisfaction. Describing was mildly and positively correlated with happiness, and weakly and positively correlated with life satisfaction. Both observing and acting with awareness were not associated with happiness and life satisfaction. Instead, nonjudging was weakly positively correlated with happiness, but no correlation has been found between this mindfulness skill and life satisfaction.

Acting with awareness was mildly and negatively correlated with vulnerability. Nonreactivity was mildly and negatively correlated with anxiety and vulnerability. Nonjudging was mildly and negatively correlated with anxiety and anger. Nonreactivity, nonjudging, and acting with awareness were equally negatively associated with both withdrawal and volatility facets. Observing, a mindfulness skill that was associated with the “core” eudaimonic PWB components in previous analyses, was totally unrelated to all neuroticism facets. By contrast, acting with awareness was overall negatively correlated with all neuroticism facets. Describing was unrelated with four out of six neuroticism facets (i.e., anxiety, depression, anger, and immoderation) (Table 2).

**Table 2:** Relationship between the FFMQ-SF and other variables

	*M*	SD	Anx	Depr	Slf_cons	Vuln	Anger	Immod	Withdr	Volatility	NEUR	Descr	Obs	Act awa	Nonrea	Nonjud	MIND	Aut	Pers gro	Purp	Self acc	Env mast	Pos rel	Core eud	Oth eud	PWB	Sat	Hap
Anx	3.06	.82	1																									
Depr	2.26	.74	.51**	1																								
Slf_cons	2.60	.73	.20**	.23**	1																							
Vuln	2.38	.78	.47**	.40**	.33**	1																						
Anger	2.71	.92	.38**	.30**	.21**	.37**	1																					
Immod	2.72	.76	.15*	.09	.04	.13	−.05	1																				
Withdr	2.57	.62	.83**	.79**	.31**	.78**	.44**	.15*	1																			
Volatility	2.72	.58	.40**	.30**	.20**	.38**	.76**	.61**	.45**	1																		
NEUR	2.62	.49	.74**	.68**	.53**	.73**	.64**	.35**	.89**	.73**	1																	
Descr	3.50	.69	−0.06	−.08	−.27**	−.26**	−.11	−.03	−.17*	−.11	−.22**	1																
Obs	3.93	.74	.00	−.05	−.07	−.07	.03	−.11	−.04	−.05	−.06	.33**																
Act awa	3.81	.73	−.18**	−.23**	−.14*	−.37**	−.20**	−.19**	−.32**	−.28**	−.35**	.20**	.23**	1														
Nonrea	2.91	.75	−.33**	−.27**	−.02	−.30**	−.22**	−.19**	−.38**	−.30**	−.37**	.02	−.01	−.02	1													
Nonjud	2.99	.78	−.32**	−.28**	−.20**	−.23**	−.29**	.00	−.35**	−.23**	−.37**	−.02	−.12	.12	.02	1												
MIND	3.41	.37	−.37**	−.37**	−.28**	−.50**	−.34**	−.21**	−.52**	−.40**	−.56**	.57**	.49**	.61**	.42**	.44**	1											
Aut	4.38	1.01	−.17*	−.14*	−.26**	−.34**	−.15*	−.25**	−.27**	−.28**	−.35**	.42**	.19**	.29**	.09	.06	.40**	1										
Pers gro	5.04	.83	−0.06	−.22**	−.16*	−.20**	−.10	−.04	−.20**	−.11	−.21**	.31**	.32**	.28**	.06	.02	.37**	.23**	1									
Purp	4.47	.93	−0.11	−.29**	−.15*	−.25**	−.11	−.11	−.27**	−.17*	−.28**	.36**	.28**	.35**	.13	.04	.44**	.33**	.37**	1								
Self acc	4.26	.98	−.35**	−.46**	−.27**	−.32**	−.26**	−.12	−.47**	−.29**	−.48**	.24**	.08	.20**	.20**	.09	.32**	.21**	.43**	.41**	1							
Env mast	4.25	.97	−.31**	−.44**	−.21**	−.38**	−.30**	−.08	−.47**	−.29**	−.47**	.17*	.10	.22**	.04	.22**	.30**	.34**	.31**	.37**	.40**	1						
Pos rel	4.04	1.06	−.21**	−.37**	−.17*	−.26**	−.15*	−.05	−.34**	−.16*	−.32**	.15*	.05	.15*	.01	.22**	.24**	.08	.24**	.24**	.34**	.36**	1					
Core eud	4.63	.68	−.16*	−.29**	−.26**	−.37**	−.17*	−.19**	−.34**	−.26**	−.39**	.50**	.35**	.41**	.13	.06	.56**	.74**	.69**	.77**	.47**	.47**	.25**	1				
Oth eud	4.18	.76	−.38**	−.55**	−.28**	−.42**	−.31**	−.11	−.56**	−.32**	−.55**	.25**	.10	.25**	.11	.24**	.38**	.27**	.43**	.45**	.76**	.76**	.76**	.51**	1			
PWB	4.41	.63	−.32**	−.49**	−.31**	−.45**	−.28**	−.17*	−.52**	−.33**	−.55**	.42**	.25**	.38**	.13	.17*	.53**	.57**	.63**	.69**	.71**	.72**	.60**	.85**	.89**	1		
Sat	4.45	1.41	−.24**	−.39**	−.15*	−.20**	−.21**	−.01	−.34**	−.18**	−.33**	.18**	.02	.11	.21**	.06	.24**	.07	.29**	.39**	.60**	.31**	.37**	.33**	.56**	.52**	1	
Hap	4.66	1.07	−.42**	−.54**	−.22**	−.32**	−.27**	.00	−.53**	−.21**	−.48**	.23**	.13	.13	.21**	.18**	.35**	.15*	.37**	.38**	.60**	.38**	.36**	.40**	.59**	.57**	.61**	1

*Anx* anxiety, *Depr* depression, *Slf*_*cons* self consciousness, *Vuln* vulnerability, *Immod* immoderation, *Withdr* withdrawal, *NEUR* neuroticism, *Descr* describing, *Obs* observe, *Act awa* acting with awareness, *Nonrea* nonreactivity, *NonJud* nonjudging, *MIND* mindfulness, *Aut* autonomy, *Pers Gro* personal growth, *Purp* purpose in life, *Self acc* self-acceptance, *Env mast* environmental mastery, *Pos rel* positive relations with others, *Core eud* “core” eudaimonic PWB aspect, *Oth eud* “other” eudaimonic PWB aspect, *PWB* psychological well-being, *Sat* life satisfaction, *Hap* subjective happiness

**p < .01; *p < .05

General mindfulness mediated between all three neuroticisms measures and both the PWB total score and its “core” eudaimonic component. By contrast, general mindfulness mediated only between volatility and life satisfaction, subjective happiness, and the “other” eudaimonic PWB component (see Table 3). A different pattern emerged when considering specific mindfulness skills as mediators. As it regards PWB total score and the PWB “core” eudaimonic component, acting with awareness was the only skill that mediated the effect of all neuroticisms measures, while nonreactivity, nonjudging, and observing were never significant. Moreover, describing mediated the relationships of broad neuroticism and withdrawal with all well-being outcomes. Nonjudging was a significant mediator of volatility with subjective happiness and the “other” eudaimonic PWB component, while nonreactivity mediated the relationship of volatility with subjective happiness and life satisfaction. Taken together, the analyses pointed out that the mediating role of acting with awareness was stronger and more consistent with the PWB measures and in particular with the “core” eudaimonic component. Furthermore, describing was a consistent mediator in the relationships of broad neuroticism and withdrawal across all well-being outcomes.

**Table 3:** Summary of regression-based mediation analyses

Mediators in the model				Effect size measures					
	Point estimates and confidence intervals of indirect effects			Model’s *R* ^2^			Indirect to direct effects ratio		
	Independent variables			Independent variables			Independent variables		
	Neuroticism	Withdrawal	Volatility	Neuroticism	Withdrawal	Volatility	Neuroticism	Withdrawal	Volatility
				Dependent variable: PWB total score					
Describing	*−.07 (−.01, −.03)*	*−.05 (−0.10, −0.01)*	−.04 (−.10, .02)	.43	.43	.34	.11	.09	.11
Nonreactivity	.01 (−.05, .09)	.01 (−.04, .07)	−.03 (−.08, .02)				−.02	−.02	.07
Nonjudging	−.01 (−.07, .06)	−.01 (−.06, .04)	−.03 (−.08, .00)				.01	.02	.09
Observing	−.01 (−.04, .01)	.00 (−.03, .01)	−.01 (−.04, .01)				.01	.01	.01
Acting with awareness	*−.07 (−.15, −.00)*	*−.05 (−.11, −.01)*	*.07 (−.13, −.02)*				.09	.10	.19
Mindfulness	*−.24 (−.37, −.12)*	*−.19 (−.28, −.10)*	*.21 (−.31, −.12)*	.37	.37	.30	.34	.35	.57
				Dependent variable: “core” eudaimonic PWB					
Describing	*−.11 (−.19, −.04)*	*−.07 (−.13, −.02)*	−.05 (−.12, .02)	.42	.41	.40	.20	.18	.16
Nonreactivity	−.03 (−.10, .03)	−.03 (−.08, .02)	−.04 (−.09, .00)				.05	.07	.12
Nonjudging	.01 (−.06, .08)	.00 (−.05, .06)	−.01 (−.05, .02)				−.02	−.01	.02
Observing	−.02 (−.06, .01)	−.01 (−.04, .01)	−.01 (−.05, .02)				.03	.02	.03
Acting with awareness	*−.12 (−.22, −.05)*	*−.09 (−.16, −.04)*	*.09 (−.16, −.04)*				.22	.24	.30
Mindfulness	*−.39 (−.55, −.27)*	*−.29 (−.40, −.20)*	*.26 (−.38, −.16)*	.42	.41	.40	.72	.79	.84
				Dependent variable: “other” eudaimonic PWB					
Describing	*−.04 (−.10, −.00)*	*−.03 (−.08, −.00)*	−.03 (−.09, .01)	.34	.35	.19	.05	.04	.07
Nonreactivity	.05 (−.04, .18)	.05 (−.03, .15)	−.02 (−.09, .06)				−.06	−.07	.04
Nonjudging	−.03 (−.10, .06)	−.02 (−.08, .05)	*.05 (−.12, −.01)*				.03	.03	.13
Observing	.00 (−.03, .01)	.00 (−.02, .01)	.00 (−.03, .01)				.00	.00	.00
Acting with awareness	−.01 (−.10, .07)	−.01 (−.08, .05)	−.05 (−.12, .01)				.01	.02	.11
Mindfulness	−.09 (−.23, .10)	−.08 (−.19, .05)	*−.16 (−.27, −.06)*	.31	.32	.18	.10	.11	.37
				Dependent variable: SWLS					
Describing	*−.09 (−.22, −.01)*	*−.06 (−.15, −.00)*	−.05 (−.15, .01)	.14	.15	.09	.09	.07	.11
Nonreactivity	−.11 (−.31, .07)	−.08 (−.25, .06)	*.13 (−.30, −.02)*				.12	.10	.31
Nonjudging	.05 (−.08, .22)	.04 (−.06, .18)	−.01 (−.11, .06)				−.06	−.05	.03
Observing	.01 (−.01, .09)	.00 (−.01, .06)	.01 (−.01, .08)				−.01	−.01	−.01
Acting with awareness	.00 (−.15, .18)	.00 (−.11, .13)	−.04 (−.16, .07)				.00	.00	.09
Mindfulness	−.12 (−.42, .17)	−.09 (−.31, .12)	*.19 (−.38, −.04)*	.11	.12	.06	.13	.12	.44
				Dependent variable: SHS					
Describing	*−.06 (−.14, −.01)*	*−.04 (−.10, −.01)*	−.04 (−.11, .01)	.26	.31	.15	.05	.04	.10
Nonreactivity	−.04 (−.18, .09)	−.01 (−.12, .09)	*.10 (−.23, −.01)*				.04	.01	.26
Nonjudging	−.04 (−.16, .07)	−.02 (−.11, .06)	*.07 (−.17, −.02)*				.03	.02	.18
Observing	−.01 (−.08, .01)	−.01 (−.05, .01)	−.01 (−.07, .01)				.01	.01	.02
Acting with awareness	.06 (−.05, .19)	.05 (−.02, .15)	−.02 (−.10, .06)				−.05	−.05	.04
Mindfulness	−.15 (−.37, .06)	−.10 (−.25, .06)	*−.24 (−.39, −.11)*	.24	.29	.13	.14	.11	.59

Point estimates are unstandardized regression coefficients based on bias-corrected bootstrap confidence interval after 10,000 bootstrap resamplings. Point estimates in italics are significant because the confidence intervals are entirely above or entirely below zero. Lower and upper limits of the bootstrap confidence intervals are in parenthesis

*SHS* Subjective Happiness Scale, *SWLS* Satisfaction with Life Scale, *PWB* psychological well-being

The data reported in Table 3 revealed other common threads. The indices of explained variance were much larger for the multiple mediation analyses than for the simple mediation analyses, but only for the PWB total score, and especially for the “core” eudaimonic component. The withdrawal aspect of neuroticism through mindfulness facets accounted for a relatively larger proportion of well-being than the volatility aspect in all analyses. A noteworthy exception was for the analyses of the “core” eudaimonic PWB components, where the variance accounted for both aspects of neuroticism was approximately the same.

Other common threads emerged when considering indirect to direct effect sizes in mediation analyses (see Table 3). Different from model’s *R* ^2^, which represents an index of total explained variance, the ratio of indirect to direct effects takes into account the unique variance accounted for by each mindfulness facet. The analyses involving the volatility aspect had a larger proportion of the direct effect accounted for by the indirect effect for all well-being outcomes. The mediating role of mindfulness constructs, especially acting with awareness, nonjudging, and nonreactivity, was relatively more important as an account for why people high on volatility, reporting a lesser use of the aforementioned skills, also reported lesser well-being.

Analysis of variance showed a main effect of gender on nonreactivity, *F*(1, 151) = 7.92, *p =* .006, and observing, *F*(1, 151) = 4.43, *p =* .037. Men had higher nonreactivity scores (*M* = 16.12, SD = 3.56) than women (*M* = 13.75, SD = 3.62); by contrast, women had higher observing scores (*M* = 16.04, SD = 2.94) than men (*M* = 14.76, SD = 3.12). The observing score displayed a significant Sex by Age interaction, *F*(1, 165) = 6.99, *p =* .009. An analysis of simple effects revealed that older women attained significantly higher scores on observing (*M* = 16.57, SD = .40) than did men in the same-age condition (*M* = 15.62, SD = .37), *F* = 13.56, *p = <*.001. The acting with awareness score displayed a significant Sex by Education Level interaction, *F* = 5.22, *p =* .024. Tests of simple effects revealed that men with a college degree had significantly higher scores on acting with awareness (*M* = 19.78, SD = .72) than did men with high school degrees (*M* = 17.54, SD = .76), *F* = 4.56, *p =* .034.

## Discussion

The present study provided preliminary evidence that mindfulness facets were differently and uniquely associated with neuroticism and well-being aspects. Moreover, different mindfulness skills represented the intervening variables by which withdrawal and volatility were related to hedonic and eudaimonic well-being. No previous study has examined these relationships based on a facet-level analysis for both mindfulness and neuroticism with PWB and SWB.

Mindfulness was strongly related to PWB (e.g., Baer et al. 2008). Our findings provided additional primary evidence suggesting that mindfulness was more strongly associated with the “core” eudaimonic components of PWB than with the “other” eudaimonic components. This finding is consistent with previous studies that proposed mindfulness as a characteristic of individuals engaged in a eudaimonic life (Ryan et al. 2008). At the facet level, describing and acting with awareness were positively correlated with all the PWB facets, and in particular with the “core” eudaimonic component of PWB. A previous study found a negative relationship between awareness-based facets, such as describing and acting with awareness, and alcohol use (Fernandez et al. 2010). Likewise, we provided preliminary evidence supporting the potential facilitating role of these specific skills for PWB. Moreover, the strongest correlation between describing and autonomy seemed to indicate that the tendency to labeling internal experiences with words was particularly relevant for regulating behavior from within as well as to resist social pressures.

Let’s now turn to the distinction between the two PWB components. The “core” eudaimonic component refers to the capacity to acquire a conceptual understanding of complex qualities in one’s life (i.e., one’s own individuality, one’s development, and one’s connections with values and meanings). By contrast, the “other” eudaimonic component, like self-acceptance, environmental mastery, and positive relations with others, is more directly related to the experience of feelings. Acting with awareness, describing, and observing were positively correlated with the “core” eudaimonic PWB component, but not with the “other” component. Our results have provided initial evidence that observing was particularly relevant for knowing how people may acquire a deeper conceptual understanding of one’s life. In fact, observing was not associated with the “other” eudaimonic components of PWB. Observing may promote well-being by adding clarity to the current experience, and facilitating a closer sensory contact with life through attention deployment, without describing that experience with words (e.g., Brown et al. 2007). In sum, our work expanded on previous research by showing that describing, observing, and acting with awareness were more relevant for experiencing a greater sense of growth and human fulfillment, namely the core aspects of positive functioning (Ryff 2014).

Another distinctive characteristic of our study was the inclusion of SWB measures. SWB differs from PWB: the former was more focused on affective and cognitive well-being aspects, the latter was more oriented toward positive functioning (Huta and Waterman 2014). Our study contributes to foster this distinction, as mindfulness facets exhibited different patterns of relationships with SWB. First, we found that observing and acting with awareness were not associated with any of the hedonic well-being measures, which, on the other hand, were intimately linked to broad neuroticism and its facets (e.g., Lauriola and Iani 2015, 2016, Lauriola and Lani 2016). Second, describing and nonreactivity were the only mindfulness skills significantly correlated with both the SWB measures. This result provided preliminary evidence suggesting that the tendency to label internal experiences (e.g., feelings, thoughts, bodily sensations) with words, as well as a non-reactive mindset, were important for experiencing pleasant emotions and for evaluating one’s life positively.

Mindfulness was strongly negatively associated with neuroticism. This result was as large as in a previous study and provided further evidence that neuroticism can inhibit mindfulness (Brown and Ryan 2003). Moreover, neuroticism facets typically related to withdrawal (e.g., anxiety, depression, and vulnerability) were associated with nonreactivity, nonjudging, and acting with awareness. This result is consistent with previous studies showing that these specific skills were related to worry and psychological symptoms (Baer et al. 2006; Fisak and Von Lehe 2012). Individuals with a non-evaluative and non-reactive mindset, together with a present-moment awareness, may tend to notice internal negative experiences of worries, depression, and vulnerability, and accept them without useless and counterproductive attitudes or reactions to them. Our findings provided preliminary evidence that also anger, a major volatility facet, exhibited the same pattern of correlation with nonreactivity, nonjudging, and acting with awareness. In other words, people high on these mindfulness facets may tend to appraise potentially upsetting internal or external events without feeling obliged to react.

According to previous studies, observing and describing were not associated with anxiety/worry symptoms (e.g., Fisak and Von Lehe 2012). Indeed, no matter how individuals may notice or describe their internal negative experiences to alleviate the associated discomfort; rather, the “how” of mindfulness did matter. Instead, a potential new finding was established: no relationships of describing and observing to anxiety and depression. Observing was totally unrelated to broad neuroticism (see also Baer et al. 2006), as well as to subjective well-being measures.

Mediation analyses led to the following conclusions. First, acting with awareness, like general mindfulness, mediated the effect of all neuroticism constructs on eudaimonic well-being outcomes (i.e., total and “core” eudaimonic PWB). This finding reinforced the view that mindfulness, and especially acting with awareness, was important for experiencing a greater sense of growth and purpose in life. In particular, the effect sizes resulting from the analyses with acting with awareness as a mediator were stronger for volatility than for withdrawal and broad neuroticism too. We interpreted these findings considering that disinhibition, a characteristic of volatility based on “acting without awareness,” might conduce to lower PWB. Conversely, the opposite of volatility, namely acting with awareness, might serve as a process to mitigate the effects of anger and immoderation as manifestations of impulsivity associated with mood-dependent behaviors.

Second, describing had consistent mediation effects across all well-being measures, but only for broad neuroticism and the withdrawal aspect. Individuals who were high on the aforementioned neuroticism measures were also reporting lesser ability to apply verbal labels to their feelings, as well as lower psychological and subjective well-being. The consistent role of describing as one of the likely processes through which neurotic individuals, and especially those disposed toward internalizing psychopathology, are less happy, satisfied with life, and less striving to realize their own true potential, can be interpreted as reflecting maladaptive coping and emotion regulation strategies. Internalizing individuals that failed to acknowledge and describe their private experience may not recognize and comprehend the mechanisms that activate and maintain the distressful experience, thus making it difficult to effectively deal with anxiety and depression. Individuals high on describing skills were able to distinguish between thoughts and feelings on the one hand and facts or environmental events on the other hand (Linehan 2014). The ability to find words to describe internal and external experiences is essential for self-regulating emotions and may represent a sort of emotion regulation process by which neuroticism is mediated. This statement is consistent with the view that describing may act as a mindful emotion regulation process that represents the capacity to stay mindfully present and aware in all occasions while letting go any emotion that is experienced (Chambers et al. 2009).

Third, nonreactivity and nonjudging were more strongly associated with neuroticism than with psychological well-being, subjective happiness, and life satisfaction. As a result, these facets of mindfulness did not mediated neuroticism and withdrawal. Occasional mediation effects for nonjudging and nonreactivity were found only between volatility and both the subjective well-being measures and the “other” eudaimonic PWB component. We speculate that the relationships of volatility with unhappiness and dissatisfaction with life might be partly due to the tendency, for individuals high on anger or immoderation, to take a negative evaluative stance (or reaction) toward thoughts and feelings, which might generate low subjective well-being. Individuals high on nonjudging skills do not evaluate something as good or bad but, instead, can observe the real nature and the effects of internal states as well as the consequences of behaviors and events and, eventually, can change such behaviors and events (Linehan 2014).

At a more general level, our mediation analyses can be interpreted in the light of separate patterns for the “what” and the “how” skills of mindfulness. In particular, acting with awareness and describing are deemed central facets of the “what” skills (Eisenlohr-Moul et al. 2012; Linehan 2014). In our study, both acting with awareness and describing yielded a very consistent pattern of indirect effects. For instance, both accounted for lesser eudaimonic well-being of neurotic individuals. Moreover, describing accounted for lesser well-being of people disposed toward internalizing tendencies. By contrast, nonjudging and nonreactivity, two facets reflecting the “how” skills, were less likely to account for eudaimonic well-being in neurotic individuals. Instead, our findings provided preliminary evidence that the “how” skills might be more closely associated with lower subjective well-being for people high on volatility. From this lens, indirect effects documented at the facet-level analysis might disclose potential clinical implications that we address in the final remarks.

Previous research with FFMQ has extensively studied sociodemographic variables of age, gender, and education (e.g., Bränström et al. 2011; Josefsson et al. 2011). Our study has examined the interaction effects between different sociodemographic factors on specific mindfulness skills scores. Men had higher nonreactivity scores than women. This finding is consistent with the view that men have a higher tendency to accept and let go of negative images, thoughts, and feelings than women (e.g., Bränström et al. 2011; Fisak and Von Lehe 2012). Compared to women, men might have a different awareness of distressing thoughts or images in which qualities of acceptance, decentering, and letting-go overcome the disposition to identify “true reality” or one’s “self” with the content of one’s thoughts (Frewen et al. 2008). Moreover, women had higher observe scores than men. This finding is consistent with previous research showing that women paid more careful attention than men to internal and external stimuli, including smells, sounds, physical sensations, colors, and shapes (Didonna and Bosio 2012; Lilja et al. 2011). A more fine-grained analysis provided initial evidence suggesting that older women had higher observing scores than older men. Finally, men with a college degree had higher acting with awareness scores than men with lower education level. Research has shown similar results regarding positive relationships between age and observing on the one hand (Lilja et al. 2011), as well as acting with awareness and education on the other hand (e.g., Bränström et al. 2011).

Although studies have shown the stability of personality and personality-well-being relationships (Steel et al. 2008), mindfulness skills are malleable and can be trained (Linehan 2014). For instance, the effects of acceptance and commitment therapy (ACT) and cognitive therapy (CT) on treatment outcomes were mediated only by specific “what” skills (i.e., acting with awareness for the ACT intervention, and observing and describing for CT; Forman et al. 2007).

Since our data provided preliminary evidence suggesting that mindfulness facets were differential mediators of neuroticism’s links to well-being, potential targets for clinical interventions might be highlighted. A training aimed at developing specific skills of mindfulness might be more effective in enhancing well-being for people high on neuroticism. As an example, anxious and depressed individuals can learn to describe thoughts and feelings and acting with awareness, in turn enhancing their ability to deal with internal and external sources of stress, to reduce the likelihood of relapse, and to improve well-being. The ability to act with awareness, entering completely into the activities in the present moment, can be enhanced by targeted interventions (e.g., DBT) to promote psychological well-being in individuals high on withdrawal. The “how” skills of mindfulness (e.g., nonjudging and nonreactivity), instead, might be enhanced to improve subjective well-being for neurotic individuals tending to acting hastily under negative mood and for those tending to get easily irritated. In sum, the relationships between neuroticism aspects with hedonic and eudaimonic well-being measures via mindfulness might then suggest which unique mindfulness skills could be prioritized for targeting clinical interventions aimed to promote well-being and to reduce psychological distress (e.g., Haenen et al. 2016; Vinci et al. 2016). Since our study has been conducted with a community-based sample, our findings provided preliminary evidence suggesting that also laypersons high on neuroticism can improve their well-being through incorporating mindful awareness practices into their everyday lives and coping repertoires. As to this point, self-help interventions had significant positive effects compared to control conditions on levels of mindfulness/acceptance, depression, and anxiety, suggesting that mindfulness and acceptance can also be learnt by self-help in a non-clinical population (Cavanagh et al. 2014).

## Limitations and Future Directions

Some limitations of the study need to be considered. First, our findings come from a cross-sectional survey, which can establish only covariation between variables in the mediation model. Although theory supports a model of neuroticism-well-being relationship via mindfulness, we cannot rule out that psychological well-being might lead to mindfulness, nor that mindfulness might prevent from becoming emotionally unstable. These alternative explanations are a challenge for future research that could be examined assuming mindfulness skills as outcomes, or predictors, based on a sequence of clinical trials or longitudinal studies.

Second, our sampling method was not random, and participants were not representative of the Italian population. However, the specific sample characteristics (i.e., middle-aged sample with relatively high education level and strong learning needs) might have ensured a better understanding and acceptance of questionnaire items, and facilitated the assessment of mindfulness skills and related constructs. Nevertheless, future studies with probabilistic sampling procedures, with larger samples, and with clinical patients will be useful to avoid bias in estimating FFMQ-SF scores and cross-validate our findings.

Third, the correlation coefficients among all facets were weak to mild (except for describing and autonomy from a facet-level analysis, and for mindfulness with neuroticism and psychological well-being from a more general level of analysis). However, the explained variance of well-being outcomes in multiple mediation analyses was sufficiently large, thus showing that lower correlations for specific measures used in this study conveyed small but unique amount of information. In a similar vein, some scales had low reliability coefficients. However, coefficients around .60 are deemed acceptable especially if the scales are very short and their validity is satisfactory (Loewenthal 2004). Exceptions to this were for the self-consciousness, environmental mastery, and purpose in life facets whose values were closer to .50 than .60. These scales were not used in mediation analyses at the facet-level.

Notwithstanding these limitations and cautions in interpreting the results of mediation analyses, we remind that one “should not let the limitations of our data collection efforts constrain the tools we bring to the task of trying to understand what our data might be telling us about the processes we are studying” (Hayes 2013, p. 17). At a more general level, our theoretical model, together with our data, seems consistent with the view that “not all the mindfulness skills are created equal.” Our findings indicate that studying interconnections among different aspects of neuroticism, mindfulness, and well-being provides some unique information that might be otherwise concealed by analyzing the data only at a broad level. Thus, we recommend that future research will deal with those interconnections looking at the three broad concepts as hierarchical constructs acting at multiple levels of analysis, a strategy that might shed light on common and peculiar associations. Taken together, we believe that our conclusions are sufficiently robust to highlight potential pathways that specifically link withdrawal and volatility to eudaimonic and hedonic well-being via mindfulness skills. In turn, these pathways indicate avenues for future clinical interventions aimed at promoting adults’ well-being, by targeting multiple distinct neuroticism to mindfulness to well-being relationships.

## References

[CR1] Albuquerque I, de Lima MP, Matos M, Figueiredo C (2012). Personality and subjective well-being: what hides behind global analyses?. Social Indicators Research.

[CR2] Anglim J, Grant S (2016). Predicting psychological and subjective well-being from personality: incremental prediction from 30 facets over the big 5. Journal of Happiness Studies.

[CR3] Baer RA, Smith GT, Hopkins J, Krietemeyer J, Toney L (2006). Using self-report assessment methods to explore facets of mindfulness. Assessment.

[CR4] Baer RA, Smith GT, Lykins E, Button D, Krietemeyer J, Sauer S, Williams JMG (2008). Construct validity of the five facet mindfulness questionnaire in meditating and nonmeditating samples. Assessment.

[CR5] Barnes SM, Lynn SJ (2010). Mindfulness skills and depressive symptoms: a longitudinal study. Imagination, Cognition and Personality.

[CR6] Bauer JJ, McAdams DP, Sakaeda AR (2005). Interpreting the good life: growth memories in the lives of mature, happy people. Journal of Personality and Social Psychology.

[CR7] Bohlmeijer E, ten Klooster PM, Fledderus M, Veehof M, Baer R (2011). Psychometric properties of the five facet mindfulness questionnaire in depressed adults and development of a short form. Assessment.

[CR8] Bränström R, Duncan LG, Moskowitz JT (2011). The association between dispositional mindfulness, psychological well-being, and perceived health in a Swedish population-based sample. British Journal of Health Psychology.

[CR9] Brown KW, Ryan RM (2003). The benefits of being present: mindfulness and its role in psychological well-being. Journal of Personality and Social Psychology.

[CR10] Brown KW, Ryan RM, Creswell JD (2007). Mindfulness: theoretical foundations and evidence for its salutary effects. Psychological Inquiry.

[CR11] Cavanagh K, Strauss C, Forder L, Jones FW (2014). Can mindfulness and acceptance be learnt by self-help?: a systematic review and meta-analysis of mindfulness and acceptance-based self-help interventions. Clinical Psychology Review.

[CR12] Chambers R, Gullone E, Allen NB (2009). Mindful emotion regulation: an integrative review. Clinical Psychology Review.

[CR13] Cohen J (1988). Statistical power analysis for the behavior science.

[CR14] Costa PT, McCrae RR (1987). Neuroticism, somatic complaints, and disease: is the bark worse than the bite?. Journal of Personality.

[CR15] DeYoung CG, Quilty LC, Peterson JB (2007). Between facets and domains: 10 aspects of the Big Five. Journal of Personality and Social Psychology.

[CR16] Didonna F (2009). Clinical handbook of mindfulness.

[CR17] Didonna F, Bosio V (2012). Misurare le abilità di mindfulness: Uno studio di validazione della versione Italiana del five facet mindfulness questionnaire [Assessing mindfulness skills: a validation study of the Italian version of the five facet mindfulness questionnaire]. Psicoterapia Cognitiva e Comportamentale.

[CR18] Diener ED, Emmons RA, Larsen RJ, Griffin S (1985). The satisfaction with life scale. Journal of Personality Assessment.

[CR19] Diener E, Suh EM, Lucas RE, Smith HL (1999). Subjective well-being: three decades of progress. Psychological Bulletin.

[CR20] Eisenlohr-Moul TA, Walsh EC, Charnigo RJ, Lynam DR, Baer RA (2012). The “What” and the “How” of dispositional mindfulness: using interactions among subscales of the five-facet mindfulness questionnaire to understand its relation to substance use. Assessment.

[CR21] Erb S, Farmer A, Mehlenbeck R (2013). A condensed dialectical behavior therapy skills group for binge eating disorder: overcoming winter challenges. Journal of Cognitive Psychotherapy.

[CR22] Fernandez AC, Wood MD, Stein LAR, Rossi JS (2010). Measuring mindfulness and examining its relationship with alcohol use and negative consequences. Psychology of Addictive Behaviors.

[CR23] Fisak B, Von Lehe AC (2012). The relation between the five facets of mindfulness and worry in a non-clinical sample. Mindfulness.

[CR24] Forman EM, Herbert JD, Moitra E, Yeomans PD, Geller PA (2007). A randomized controlled effectiveness trial of acceptance and commitment therapy and cognitive therapy for anxiety and depression. Behavior Modification.

[CR25] Frewen PA, Evans EM, Maraj N, Dozois DJ, Partridge K (2008). Letting go: mindfulness and negative automatic thinking. Cognitive Therapy and Research.

[CR26] Giluk TL (2009). Mindfulness, big five personality, and affect: a meta-analysis. Personality and Individual Differences.

[CR27] Grossman P, Van Dam NT (2011). Mindfulness, by any other name…: trials and tribulations of sati in western psychology and science. Contemporary Buddhism.

[CR28] Haenen S, Nyklíček I, van Son J, Pop V, Pouwer F (2016). Mindfulness facets as differential mediators of short and long-term effects of mindfulness-based cognitive therapy in diabetes outpatients: findings from the DiaMind randomized trial. Journal of Psychosomatic Research.

[CR29] Hampson SE (2012). Personality processes: mechanisms by which personality traits “get outside the skin”. Annual Review of Psychology.

[CR30] Hayes AF (2013). Introduction to mediation, moderation, and conditional process analysis: a regression-based approach.

[CR31] Huta V, Joseph S (2015). The complementary roles of eudaimonia and hedonia and how they can be pursued in practice. Positive psychology in practice: promoting human flourishing in work, health, education, and everyday life.

[CR32] Huta V, Waterman AS (2014). Eudaimonia and its distinction from hedonia: developing a classification and terminology for understanding conceptual and operational definitions. Journal of Happiness Studies.

[CR33] Iani L, Lauriola M, Layous K, Sirigatti S (2014). Happiness in Italy: translation, factorial structure and norming of the subjective happiness scale in a large community sample. Social Indicators Research.

[CR34] Johnson JA (2014). Measuring thirty facets of the five factor model with a 120-item public domain inventory: development of the IPIP-NEO-120. Journal of Research in Personality.

[CR35] Josefsson T, Larsman P, Broberg AG, Lundh LG (2011). Self-reported mindfulness mediates the relation between meditation experience and psychological well-being. Mindfulness.

[CR36] Kabat-Zinn J (1994). Wherever you go, there you are: mindfulness meditation in everyday life.

[CR37] Keyes CL, Shmotkin D, Ryff CD (2002). Optimizing well-being: the empirical encounter of two traditions. Journal of Personality and Social Psychology.

[CR38] Lauriola M, Iani L (2015). Does positivity mediate the relation of extraversion and neuroticism with subjective happiness?. PLoS One.

[CR39] Lauriola M, Lani L (2016). Personality, positivity and happiness: a mediation analysis using a bifactor model. Journal of Happiness Studies.

[CR40] Lilja, J. L., Frodi-Lundgren, A., Hanse, J. J., Josefsson, T., Lundh, L. G., Sköld, C., … & Broberg, A. G. (2011). Five facets mindfulness questionnaire—reliability and factor structure: a Swedish version. *Cognitive Behaviour Therapy, 40*(4), 291-303. 10.1080/16506073.2011.580367 21770845

[CR41] Linehan MM (2014). DBT® skills training manual.

[CR42] Loewenthal KM (2004). An introduction to psychological tests and scales.

[CR43] Lyubomirsky S, Lepper HS (1999). A measure of subjective happiness: preliminary reliability and construct validation. Social Indicators Research.

[CR44] Ormel, J., Oldehinkel, A. J., Ferdinand, R. F., Hartman, C. A., de Winter, A. F., Veenstra, R., … & Verhulst, F. C. (2005). Internalizing and externalizing problems in adolescence: general and dimension-specific effects of familial loadings and preadolescent temperament traits. *Psychological Medicine*, *35*(12), 1825–1835. 10.1017/S0033291705005829 16300695

[CR45] Paulik G, Simcocks A, Weiss L, Albert S (2010). Benefits of a 12-week mindfulness group program for mental health consumers in an outpatient setting. Mindfulness.

[CR46] Roberts BW, Walton KE, Viechtbauer W (2006). Patterns of mean-level change in personality traits across the life course: a meta-analysis of longitudinal studies. Psychological Bulletin.

[CR47] Ruini C, Ottolini F, Rafanelli C, Ryff CD, Fava GA (2003). La validazione italiana delle Psychological Well-Being Scales (PWB). Rivista di Psichiatria.

[CR48] Ryan RM, Huta V, Deci EL (2008). Living well: a self-determination theory perspective on eudaimonia. Journal of Happiness Studies.

[CR49] Ryff CD (2014). Psychological well-being revisited: advances in the science and practice of eudaimonia. Psychotherapy and Psychosomatics.

[CR50] Ryff CD, Keyes CLM (1995). The structure of psychological well-being revisited. Journal of Personality and Social Psychology.

[CR51] Sirigatti S, Penzo I, Iani L, Mazzeschi A, Hatalskaja H, Giannetti E, Stefanile C (2013). Measurement invariance of Ryff’s psychological well-being scales across Italian and Belarusian students. Social Indicators Research.

[CR52] Steel P, Schmidt J, Shultz J (2008). Refining the relationship between personality and subjective well-being. Psychological Bulletin.

[CR53] Veehof MM, ten Klooster PM, Taal E, Westerhof GJ, Bohlmeijer ET (2011). Psychometric properties of the Dutch Five Facet Mindfulness Questionnaire (FFMQ) in patients with fibromyalgia. Clinical Rheumatology.

[CR54] Vinci C, Spears CA, Peltier MR, Copeland AL (2016). Facets of mindfulness mediate the relationship between depressive symptoms and smoking behavior. Mindfulness.

